# Coping flexibility in college students with depressive symptoms

**DOI:** 10.1186/1477-7525-8-66

**Published:** 2010-07-13

**Authors:** Ji-Gang Zong, Xiao-Yan Cao, Yuan Cao, Yan-Fang Shi, Yu-Na Wang, Chao Yan, John RZ Abela, Yi-Qun Gan, Qi-Yong Gong, Raymond CK Chan

**Affiliations:** 1 Neuropsychology and Applied Cognitive Neuroscience Laboratory, Institute of Psychology, Chinese Academy of Sciences, Beijing, China; 2 Key Laboratory of Mental Health, Institute of Psychology, Chinese Academy of Sciences, Beijing, China; 3 Graduate School, Chinese Academy of Sciences, Beijing, China; 4 Department of Applied Social Studies, City University of Hong Kong, Hong Kong Special Administrative Region, China; 5 Department of Psychology, Rutgers University, New Brunswick, New Jersey, USA; 6 Department of Psychology, Peking University, Beijing, China; 7 Huaxi MR Research Centre, Department of Radiology, West China Hospital/West China School of Medicine, Sichuan University, Chengdu, China

## Abstract

**Background:**

The current study explored the prevalence of depressed mood among Chinese undergraduate students and examined the coping patterns and degree of flexibility of flexibility of such patterns associated with such mood.

**Methods:**

A set of questionnaire assessing coping patterns, coping flexibility, and depressive symptoms were administered to 428 students (234 men and 194 women).

**Results:**

A total of 266 participants both completed the entire set of questionnaires and reported a frequency of two or more stressful life events (the criterion needed to calculate variance in perceived controllability). Findings showed that higher levels of depressive symptoms were significantly associated with higher levels of both event frequency (r = .368, p < .001) and event impact (r = .245, p < .001) and lower levels of perceived controllability (r = -.261, p < .001), coping effectiveness (r = -.375, p < .001), and ratio of strategy to situation fit (r = -.108, p < .05). Depressive symptoms were not significantly associated with cognitive flexibility (variance of perceived controllability; r = .031, p = .527), Gender was not a significant moderator of any of the reported associations.

**Conclusions:**

Findings indicate that Chinese university students with depressive symptoms reported experiencing a greater number of negative events than did non-depressed university students. In addition, undergraduates with depressive symptoms were more likely than other undergraduates to utilize maladaptive coping methods. Such findings highlight the potential importance of interventions aimed at helping undergraduate students with a lower coping flexibility develop skills to cope with stressful life events.

## Background

Coping refers to the "thoughts and behaviors that people use to manage the internal and external demands of situations that are appraised as stressful" [1]. A large body of research has accumulated in the past three decades [1] demonstrating that coping is a crucial determinant of psychological well-being with its outcome depending largely on the types of strategies employed [2-4]. Effective coping is achieved only if the situation has been appraised accurately and the coping strategies that are used are appropriate for the situation [5]. When the appropriate coping style is used, positive emotion can occur even when depression and distress are frequent [1]. A failure, however, of effective coping with stress may lead to psychological problems [4,6].

Coping flexibility includes 3 components: cognitive flexibility, situation-strategy fit, and goal attainment. Cognitive flexibility refers to the degree to which an individual's cognitive appraisals of controllability vary across situations. Strategy-situation fit refers to the degree to which the coping strategy used fits the nature of the situation. Goals attainment refers to the degree to which an individual's goals are attained by the coping strategies used. Higher levels of coping flexibility (i.e., high cognitive flexibility, strategy-situation fit, and goal attainment) have been found to be associated with higher levels of positive adjustment [7] and lower levels of symptoms of burnout [8]. Coping flexibility has been found to be associated with decreases in anxiety symptom severity and increases in quality of life over a two month follow-up interval [9]. Coping flexibility is also associated with a decreased likelihood of experiencing increases in depressive symptoms following the occurrence of stressful life events [10]. On the other hand, lower level of coping flexibility have been found to predict increases in depressive symptoms over time [11].

Individual differences in coping flexibility may be explained by variation in cognitive processes [3,7]. Cheng and Cheung (2005) [7] posit two cognitive processes that underlie coping flexibility: differentiation and integration. Differentiation refers to "an ability to recognize multiple dimensions embedded in a perceived domain and to the taking of different perspectives when considering the domain (p.862)". Regarding integration, although these different dimensions may be in conflict with one another, they should be evaluated and then combined or "integrated". Consistent with such hypotheses, Cheng and Cheung have found that individuals with higher levels of coping flexibility to evaluate stressful situations in terms of controllability and impact, and to employ higher levels of more monitoring in controllable situations.

It seems logical to assume that the more educated people are, the higher their cognitive ability. Gan and colleagues (2006) [5], however, reported that depressive participants with higher educational qualifications exhibited lower levels of coping flexibility. In fact, when stressful events were encountered in daily life, active-inflexible coping was the dominant strategy employed by Chinese university students perceiving most situations as controllable [12].

Undergraduate students face a number of daily life stressors placing them at high risk for developing negative affect,(i.e., depressed mood) and, in serious cases, psychological problems[4,13]. Such students are at the unique risk for experiencing student burnout, which is "a set of psychological syndromes that occurs due to chronic academic stress" [14]. Many factors have been found to impact a student's mood including availability of support from close friends and family members and economic status [15]. In female undergraduates, academic, economic and interpersonal stressors have been found to induce negative mood through the mediating role of maladaptive problem-solving strategies [16]. Chinese undergraduates with depressive symptoms have been found to utilize maladaptive coping methods [13] with depressed individuals lacking coping flexibility in all three domains (i.e., cognitive flexibility, situation-strategy fit and goal attainment) [5]. As depressive symptoms have a negative and influential impact on an individual's thinking, they can create a vicious negative feedback loop between maladaptive coping and depression [3].

The findings summarized above suggest that it is important to empower undergraduate students, especially those exhibiting depressed mood, with flexible and effective skills for coping with stress [5]. There has, however, been limited research examining coping flexibility in university students in China. The current study aimed to explore the prevalence of depressed mood among Chinese undergraduate students and to examine the coping patterns and degree of flexibility associated with such mood.

## Methods

### Participants

Participants consisted of 428 college students (234 men and 194 women) recruited from universities in Beijing. The mean age of participants was 18.49 years (SD = 0.789) and participants, on average, had completed 12.27 years (SD = 0.712) of education. Participants received $10 RMB in return for completing questionnaires. Beck Depression Inventory (BDI [17]; Chinese version, Shek [18]) scores were used to classify participants into depressed (score ≥ 14) and non-depressed (score ≤ 4) groups. A total of 266 participants both completed the entire set of questionnaires and reported a frequency of two or more stressful life events (the criterion needed to calculate variance in perceived controllability). Of these participants, 56 (30 men and 26 women) were classified as depressed (MEAN BDI = 20.03; SD = 6.80) and 210 (177 men and 93 women) as non-depressed (MEAN BDI = 1.67; SD = 1.50). In order to match the two groups in terms if demographic data, 56 non-depressed participants were randomly selected for subsequent data analysis.

### Measures

#### Coping Flexibility Questionnaire

The Coping Flexibility Questionnaire (CFQ) [19] is a questionnaire designed to assess the coping styles exhibited by individuals when encountering stressful life events. Items are rated on 6-point Likert scales. The first subscale assesses variability in perceived controllability across situation with higher scores indicating greater controllability and perceived impact. The second subscale assesses the 'goodness of fit' between coping strategies and the nature of the stressful situation encountered by asking participants to describe both the strategies use to cope with stressors and the primary goal in using such strategies (either problem-focused or emotion-focused). The third subscale assesses the perceived effectiveness of coping behaviors in attaining desired with higher ratings indicating greater perceived effectiveness. The current study utilized the Chinese version of the CFQ [12] - an adaptation of the original version in which the original open-ended question assessing the occurrence of stressful life events was changed to a pre-determined list of 40 stressful events instead (e.g., conflicts with other people over hassles, difficulties encountered in study, quarrels or breakup with girlfriend/boyfriend). This adaptation exhibits high levels of validity in discriminating students exhibiting depressive symptoms from healthy controls.

#### The Beck Depression Inventory

The Beck Depression Inventory (BDI) [17] is a 21-item, self-report questionnaire that assesses depressive symptoms experienced in the past two weeks. Scores range from 0 to 63, with higher scores indicating higher levels of depressive symptoms. The BDI has been found to exhibit high levels of reliability and validity [20]. The Chinese version of the BDI, which was used for the current study, has been found to exhibit strong reliability and criterion-related validity [18,19,21] in mainland China [22,23]

#### Procedure

The current study was approved by the ethics committee of the Institute of Psychology of the Chinese Academy of Sciences. Participants were given a general introduction to the study as well as the opportunity to ask questions about the study. Written consent was obtained prior to the administration of questionnaires. Questionnaires were administered to groups of students.

## Results

### Overview of Statistical Analyses

Data was analyzed using SPSS version 13.0. Bivariate correlation was used to examine the correlation of depressive symptoms and variables in CFQ in the sample as a whole. Pearson chi-square test was used to examine significant differences between the two groups (i.e., depressed versus non-depressed) in terms of gender distribution; Multivariate analysis of variance (MANOVA), was used to examine the impact of gender, group membership (Depressed vs. Non-depressed), gender and their interaction on the indexes of the scales. Binary Logistic regression analysis was also conducted with all indexes of questionnaires as independent variables and group status as the dependent variable. *Partial η*^*2 *^values (small, 0.2~0.3; medium, 0.5; large, 0.8 and above) were also calculated to determine the size of effects (i.e. differences between depressed group and non-depressed group). Statistical significance was set at p < 0.05.

#### Bivariate Correlations

Means, standard deviations, and the pattern of inter-correlation for all variables are presented in Table 1. Several findings warrant attention. First, higher levels of depressive symptoms were significantly associated with higher levels of event frequency (r = .368, p < .*001*) and event impact (r = .245, p < .001) and lower levels of perceived controllability (r = -.261, p < .001), coping effectiveness (r = -.375, p < .001), and ratio of strategy to situation fit (r = -.108, p < .05). At the same time, depressive symptoms were not significantly associated with cognitive flexibility (variance of perceived controllability; r = .031, p = .527), Second, with the exception of the association between perceived controllability and coping effectiveness (r = .519, p < .001), the subscale of the CFQ were only modestly (p < .20) to moderately (.20 < p < .30) associated with one another suggesting that the constructs assessed by these subscales are not only conceptually distinct but also empirically distinct. Last, gender was not a significant moderator of any of the reported associations.

**Table 1:** Means, Standard Deviations, and Inter-Correlations for all Variables

	1	2	3	4	5	6	7
1. Depressive Symptoms	-						

2. Event Frequency	.368 ***	-					

3. Event Impact	.245***	.156**	-				

4. Perceived Controllability	-.261***	-.168***	-.246***	-			

5. Cognitive Flexibility	.031	.044	.103*	-.155**	-		

6. Coping Effectiveness	-.375***	-.160**	-.233***	.519***	.076	-	

7. Ratio of Strategy to Situation Fit	-.108*	-.076	-.028	.153**	.009	.066	-

Mean	6.313	7.899	3.118	3.802	1.350	4.239	0.584

SD	6.696	6.832	1.036	1.165	1.880	1.093	0.332

* p < 0.05, ** p < 0.001, *** p < 0.0001

#### Group Comparisons

The results of a Chi square analysis indicated that the two groups (i.e. depressed and non-depressed) did not vary in terms of gender composition (*χ*^*2*^_(1, n = 112) _= 0.358, p = 0.085). The results of a multivariate analysis of variance (MANOVA) revealed a significant effect of group status on all variables (F_(18, 91) _= 13.777, p < 0.001) with the effect size being large (*partial η*^*2 *^= 0.732). At the same time, neither gender (p = 0.249) nor the interaction between gender and depressive symptoms (p = 0.348) exhibited significant effects.

As presented in Table 2. in comparison to the non-depressed group, the depressed group reported both a greater frequency of stressful life events (F_(1, 108) _= 27.387, p < 0.001, *partial η*^*2 *^= 0.202) and a greater impact of such events on themselves (F_(1, 108) _= 14.281, p < 0.001, *partial η*^*2 *^= 0.117). The depressed group perceived stressors as being more uncontrollable (F_(1, 108) _= 10.530, p = 0.002, *partial η*^*2 *^= 0.089). Although there was no significant group difference on the index of cognitive flexibility (variance of perceived controllability; p = 0.270), the depressed group reported lower levels of coping effectiveness (F_(1, 108) _= 45.754, p < 0.001, *partial η*^*2 *^= 0.298) and a lower ratio of strategy-situation fit (F_(1, 108) _= 6.273, p = 0.014, *partial η*^*2 *^= 0.055). Compared to the non-depressed group, the depressed group utilized more problem focused coping strategies in uncontrollable situations and less problem focused coping strategies in controllable situations (F_(1, 108) _= 4.880, p = 0.029, *partial η*^*2 *^= 0.043; F_(1, 108) _= 14.140, p < 0.001, *partial η*^*2 *^= 0.116, respectively). There were no significant group differences in the use of emotion focused strategies in either controllable (p = 0.369) or uncontrollable (p = 0.268) situations.

**Table 2:** Results of MANOVA for CFQ questionnaire indexes between depressive and non-depressive groups

Items	Non-depressed (n = 56)		Depressed Group (n = 56)		F	*p*	*partialη2*
				
	Mean	SD	Mean	SD			
	MANOVA				13.777	< 0.001	0.732
Frequency of stressful life events	5.536	3.139	12.661	9.681	27.448	< 0.0005	0.200
Impact of stressful life events	2.912	0.987	3.643	1.173	12.709	0.001	0.104
Perceived controllability	3.883	1.191	3.154	1.173	10.655	0.001	0.088
Variance of perceived controllability	1.163	1.325	1.567	2.498	1.114	0.287	0.010
Coping effectiveness	4.784	1.023	3.397	1.122	46.711	< 0.0005	0.298
Ratio of strategy-situation fit	0.665	0.341	0.507	0.303	6.692	0.011	0.057
Ratio of problem focused strategy in controllable situation	0.405	0.367	0.184	0.236	14.374	< 0.0005	0.116
Ratio of emotion focused strategy in controllable situation	0.164	0.282	0.217	0.283	0.970	0.327	0.009
Ratio of problem focused strategy in uncontrollable situation	0.158	0.273	0.273	0.271	4.976	0.028	0.043
Ratio of emotion focused strategy in uncontrollable situation	0.124	0.218	0.168	0.220	1.154	0.285	0.010

Note:

CFQ: The Coping Flexibility Questionnaire;

#### Examining the Unique Effects of CFQ Subscales

Given that the depressed and non-depressed groups significantly differed from one another on multiple subscales of the CFQ (i.e., event frequency, event impact, perceived controllability. coping effectiveness, and ratio of strategy-situation fit), we examined whether each of these subscales was a significant predictor of group status (i.e., 0 = non-depressed and 1 = depressed) after controlling for the other subscales which varied as a function of group status. Event frequency (B = 0.214, SE = 0.064, p = .001), event impact (B = 0.678, SE = 0.321, p < .05), coping effectiveness (B = -1.259, SE = 0.320, p < .001), and ratio of strategy-situation fit (B = -2.082, SE = 0.919, p < .05) each exhibited a unique effect in predicting group status. At the same time, perceived controllability failed to exhibit a unique effect (B = 0.157, SE = 0.294, p = .593)

## Discussion

Several findings emerge from the current study. First, consistent with prior research [24], university students with depressive symptoms reported experiencing a greater number of negative events than did non-depressed university students. The magnitude of this difference was quite large with the symptomatic group reporting approximately 2.3 times the number of negative events than the asymptomatic group. University students with depressive symptoms also reported negative events as having a greater impact on their lives suggesting that in addition to experiencing a greater frequency of negative events they also experience negative events that carry a greater degree of threat [25]. Such findings are consistent with the hypothesis that negative events play a central role in the development of depressive symptoms and highlight the importance of discovering factors that may mediate [4] or moderate [26] this association - such as coping flexibility.

Second, consistent with the results of past research [13] the results of the current study suggest that undergraduates with depressive symptoms are more likely than other undergraduates to utilize maladaptive coping methods. Consistent with models which highlight the centrality of the concept of helplessness in the etiology of depression [27-29], university students with depressive symptoms reported perceiving events as more uncontrollable than did non-depressed university students [7]. Given that coping is effective only if the coping strategies used are appropriate for the situation that has been appraised accurately [5], a negative perceptual bias towards perceiving events as uncontrollable may lead depressed individuals to selecting inappropriate coping mechanisms for the events they encounter. Consistent with models that suggest that depressed individuals utilize maladaptive coping strategies [30], depressed university students perceived their coping strategies as less effective in achieving desired outcomes (i.e., modifying situations or emotions). Last, university students with depressive symptoms were more likely to report utilizing coping strategies that do not represent a good fit for the type of event they have encountered. The pattern of findings observed in the current study is similar to those obtained in samples of depressed patients who cannot discriminate controllability and perceive situation as uncontrollable whilst using problem-focused coping less [5]. Thus, findings suggest not only do university students with depressive symptoms experience a greater frequency of negative events of greater magnitude than non-depressed university students, but they are less well-equipped to cope with such stressors once they occur.

Depression may have a cognitive basis. Hong (2007) [31]reported worry to be associated with both anxious and depressive symptoms and rumination to be associated with depressive symptoms. Worry and rumination were also found to be associated with lower perceived coping effectiveness and disengagement respectively [31]. It is likely that these two cognitive processes prevent the employment of effective coping strategies and consequently lead to an exacerbation of depressive symptoms over time. Lower levels of perceived coping effectiveness may reduce an individual's motivation to use flexible coping following stressful events [10]. Cheng (2003) [9].proposed a dual-process model of coping flexibility to capture the need for closure, influences the cognitive appraisal of the stressful situation. Need for closure refers to the tendency to rely on familiar coping strategies without considering alternatives. Those with a high need for closure have been found both to be less likely to discriminate among stressful situations and to employ flexible coping strategies. As participants in the current study with elevated levels of depressive symptoms perceived their coping strategies as less effective in achieving desired outcomes, it is likely that they were reluctant or less motivated to actively appraise the stressful situations they encountered from multiple perspectives or different dimensions. The failure to differentiate between situations in terms of the controllability dimension may have resulted in the use of maladaptive coping strategies without a good fit found among depressed participants [7]

Several limitations of the current study should be noted. First, as the current study was cross-sectional in nature, conclusions about the directions of the reported associations cannot be drawn. Longitudinal research designs are needed to determine whether coping strategies influence the subsequent development of depressive symptoms or whether the onset of depressive symptoms negatively impacts the types of coping strategies individuals employ. Second, a self-report measure was used to assess depressive symptoms. Although, the BDI exhibits high levels of reliability and validity, results cannot necessarily be generalized to clinically significant depressive episodes, Future research is likely to benefit from assessing the presence and/or absence utilizing semi-structured clinical interviews to see if the current pattern of findings extend to clinical samples. Third, a self-report measure was used to assess negative event frequency and severity as well as coping flexibility. Given that self-report measures are likely impacted by informant biases, future research is likely to benefit from assessing these constructs utilizing contextual threat interviews [25], other-reports, and behavioral observations (i.e., multi-method, multi-informant approach). Last, the current study utilized a sample of university students with non-clinical depressive symptoms. Although studying coping in university students is a topic of great significance, the current findings cannot be generalized to other populations (e.g., clinical major depression patients). Future research is thus needed examining whether the current findings replicate in community samples.

## Conclusions

In sum, undergraduate students face a number of daily life stressful events, which place them at risk of developing negative or depressive mood and even psychological problems in serious cases [4,13]. This study shows that it is important that more attention and help is given to undergraduate students with a lower coping flexibility, to empower them with the skills to cope with stressful life events. With flexible coping, positive adjustments can arise [7], and positive emotion can occur even when depression and distress are frequent [1].

## Competing interests

The authors declare that they have no competing interests.

## Authors' contributions

RCKC generated the idea and designed the study, wrote up the first draft of the paper. JZ collected and analyzed the data, wrote up the first draft of the paper. XC, YC, YS, YW, and CY collected the data and participated in the preparation of the paper. JRZA participated in the preparation of the paper. YG contributed the scale for use and commented on the first draft of the paper. All authors read and approved the final manuscript.
